# Automatic Categorization and Scoring of Solid, Part-Solid and Non-Solid Pulmonary Nodules in CT Images with Convolutional Neural Network

**DOI:** 10.1038/s41598-017-08040-8

**Published:** 2017-09-01

**Authors:** Xiaoguang Tu, Mei Xie, Jingjing Gao, Zheng Ma, Daiqiang Chen, Qingfeng Wang, Samuel G. Finlayson, Yangming Ou, Jie-Zhi Cheng

**Affiliations:** 1 School of Communication and Information Engineering, University of Electronic Science and Technology of China, Xiyuan Ave. 2006, West Hi-Tech Zone, Chengdu, Sichuan 611731 China; 2 Department and Graduate Institute of Electrical Engineering, Chang Gung University, 259 Wen-Hwa 1st Road, Kwei-Shan Tao-Yuan, 333 Taiwan; 3 School of Electronic Engineering, University of Electronic Science and Technology of China, Xiyuan Ave. 2006, West Hi-Tech Zone, Chengdu, Sichuan 611731 China; 4 Third Military Medical University, Chongqing, 400038 China; 5 School of Software Engineering, University of Science and Technology of China, 230026 Hefei, China; 6 Department of Systems Biology, Harvard Medical School, 10 Shattuck St., Boston, MA 02115 USA; 7 Harvard-MIT Division of Health Sciences and Technology (HST), 77 Massachusetts Avenue, E25-518, Cambridge, MA 02139 USA; 8 Department of Radiology, Harvard Medical School, 1 Autumn St., Boston, MA 02215 USA

## Abstract

We present a computer-aided diagnosis system (CADx) for the automatic categorization of solid, part-solid and non-solid nodules in pulmonary computerized tomography images using a Convolutional Neural Network (CNN). Provided with only a two-dimensional region of interest (ROI) surrounding each nodule, our CNN automatically reasons from image context to discover informative computational features. As a result, no image segmentation processing is needed for further analysis of nodule attenuation, allowing our system to avoid potential errors caused by inaccurate image processing. We implemented two computerized texture analysis schemes, classification and regression, to automatically categorize solid, part-solid and non-solid nodules in CT scans, with hierarchical features in each case learned directly by the CNN model. To show the effectiveness of our CNN-based CADx, an established method based on histogram analysis (HIST) was implemented for comparison. The experimental results show significant performance improvement by the CNN model over HIST in both classification and regression tasks, yielding nodule classification and rating performance concordant with those of practicing radiologists. Adoption of CNN-based CADx systems may reduce the inter-observer variation among screening radiologists and provide a quantitative reference for further nodule analysis.

## Introduction

Pulmonary nodules are small masses within the pulmonary interstitium that pose a difficult but extremely important differential diagnosis when detected by computerized tomography. In CT scans, pulmonary nodules appear as relatively bright structures, and can appear in isolation within the lung parenchyma or as an attachment to the lung chest wall, airway, pulmonary vessel, or fissure. Recent studies have suggested that the phenotypic features of pulmonary nodules detected in CT images are predictive of malignancy^1^, and are thus extremely important for determining further diagnosis, management, and treatment. In clinical practice, however, the differential diagnosis of a pulmonary nodule in CT images is a complicated and challenging decision process^2, 3^. Despite continuous modification and improvement in diagnostic guidelines over the years^3, 4^, physicians’ accuracy in diagnosing pulmonary nodules remains varied^3, 5^ and also highly dependent on experience^6^. These clinical challenges motivate the development of computer-aided diagnosis (CADx) systems that perform objective, quantitative, and consistent identification of nodule malignancy in CT images^7–9^. When successfully implemented, CADx systems are expected to improve diagnostic outcomes^6^ and reduce unnecessary biopsy, thoracotomy and surgery^10^.

**Figure 1:**
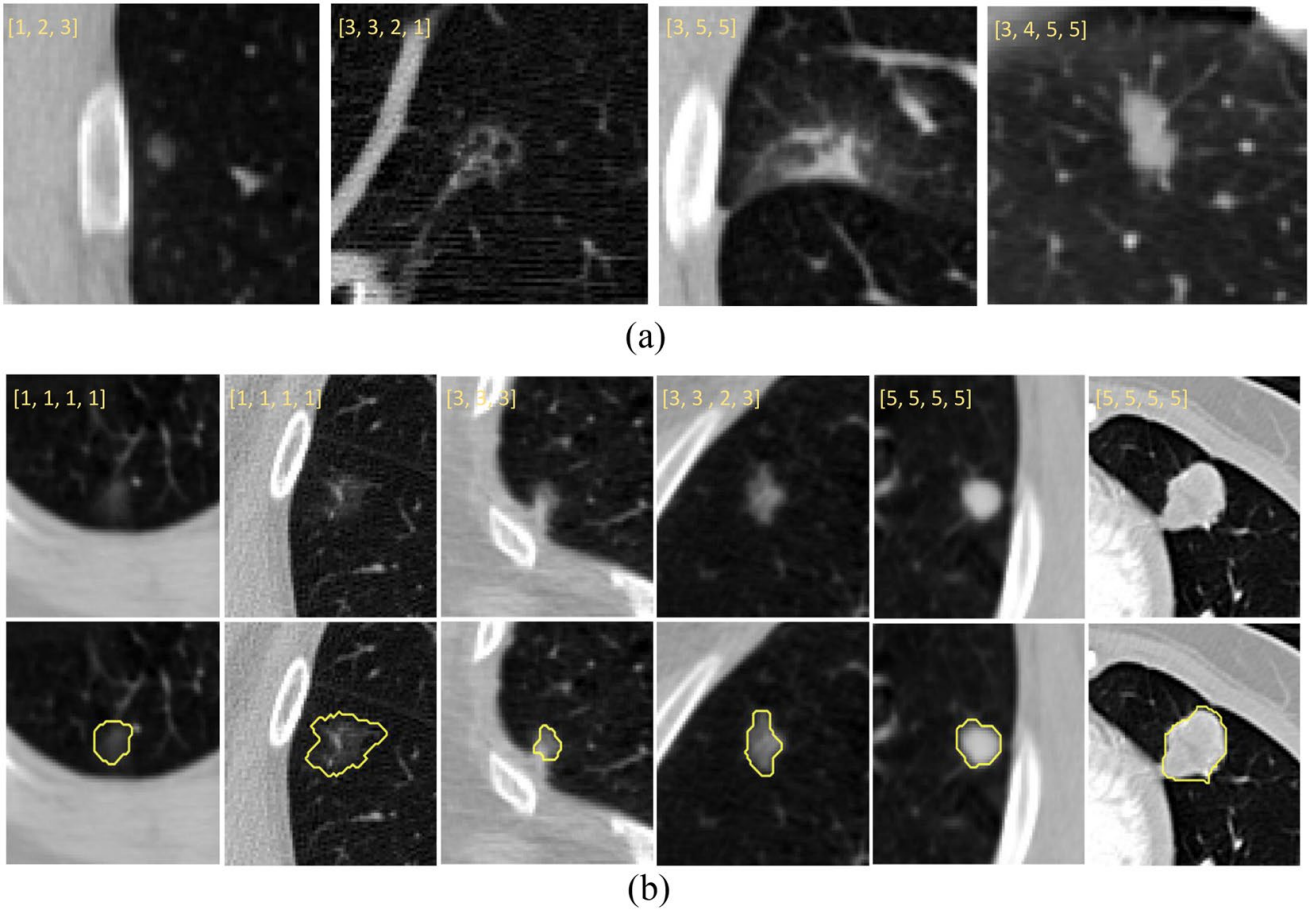
(**a**) 4 nodules with ambiguous rating/classification. Each nodule was annotated by three to four different radiologists. The annotated scores from radiologists are shown in the upper port of each nodule ROI with yellow numbers. The left most nodule was classified as non-solid by first two radiologists and part-solid by the third radiologist. The second nodule was regarded as part-solid by the first two radiologists but non-solid by the last two radiologists. The third nodule was classified as part-solid by the first radiologist whereas the second and third radiologists thought this was a solid nodule. For the fourth nodule, the second, third and fourth radiologists classified it as solid, however the first radiologist classified it as part-solid. (**b**) Illustrations of the different, possibly empty segmentations for non-solid lesions (left two columns), part-solid lesions (middle two columns), and solid lesions (right two columns). The top row shows the ROIs of the original CT images without segmentation, while the bottom row lists the corresponding images with manual segmentations.

Recent clinical studies have suggested that non-solid and especially part-solid nodules may be more likely than solid nodules to be confirmed as pulmonary adenocarcinoma^11–15^. This has prompted the Fleischner Society^3^ and others^1, 15–17^ to recommend that CT diagnostic procedures include the classification of pulmonary nodules into the categories of solid, part-solid, and non-solid nodules. However, as with the diagnosis of nodule malignancy more generally^5^, inter-observer disagreement has also been demonstrated in expert differentiation of nodules into non-solid, part-solid and solid categories^18, 19^. As can be seen in Fig. 1(a), this can be attributed in part to visual ambiguity between non-solid and part-solid nodules as well as between part-solid and solid nodules. As such, prominent works have indicated that the computerized differentiation^20^ of pulmonary nodules into the three categories may serve as an objective reference for the diagnosis and prognosis prediction^1, 13^. To date, however, little discussion has taken place regarding the development of such systems; our literature search identified only a few clinical studies, which mainly focused on subjective categorization schemes^18, 19, 21^.

Jacobs *et al*.^20^ proposed a computer-aided classification method to categorize nodules in CT images into solid, part-solid, and non-solid classes. However, the work was limited in scope, including only 50 nodules from each class with a diameter range of 6.0–28.3 mm. Furthermore, the histogram-based method that the paper employed requires image segmentation to delimit the region tightly surrounding the nodule. Since the margins of certain nodules are poorly defined, especially in the cases of the high-risk non-solid and part-solid nodules, the task of nodule segmentation is quite difficult and potentially unreliable, as shown in Fig. 1(b). Furthermore, in the context of conventional CADx schemes^12, 20^, the process of image segmentation commonly requires user intervention (e.g., manual refinement^22, 23^, candidate selection^24^, etc.) to obtain accurate contours. This requirement for user intervention results in engineering challenges, user time, and an additional source of variability. Therefore, it is unsurprising that past studies have shown the correctness of image segmentation results to be a key determinant of CADx performance^7, 24^.

In this paper, we develop a new CADx method to automatically categorize solid, part-solid and non-solid nodules in CT images, without depending on image segmentation. Our method, built using a convolutional neural network (CNN), requires as user input only a two-dimensional region of interest (2D ROI) around each nodule, and thence automatically reasons from image context to produce a nodule rating. Our CADx is built using the rich annotation resources provided by the public LIDC dataset and implements both classification (3 classes) and regression (scoring 1–5) schemes. Taken together, our work serves as a new, segmentation-free, computerized reference for nodule attenuation.

## Experiment Results

To evaluate our approach, we implemented CNN- and HIST-based methods for both classification and regression schemes. In order to facilitate an effective comparison of the two methods, we additionally implemented two slice selection strategies (SINGLE and ALL) to account for potential uncertainty with respect to the depth of each nodule. We here report results for the regression and classification tasks separately for each of the slice selection strategies, the details of which can be found in Methods.

### Non-solid, Part-solid, Solid Classification

Tables 1 and 2 report the classification performance by the CNN and HIST methods, respectively. For each method, we report the confusion matrix, accuracy, precision and recall, all computed using 10-times 10-fold cross validation to ensure robustness to data dependence. As can be observed, the accuracy of the CNN trained with slice strategy ALL (CNN-ALL) was 2% higher than the CNN with strategy SINGLE (CNN-SINGLE), and at least 18% higher than HIST with respect to the ALL and SINGLE strategies. For precision and recall metrics, the CNN-ALL outperformed the schemes of CNN-SINGLE, HIST-ALL and HIST-SINGLE.

**Table 1:** Mean ± standard deviation summary of confusion matrixes achieved by CNN model over 10 times 10-fold cross validations on the testing nodules.

The mean confusion matrix of CNN over the cross-validation of all folds				
Rad\CNN	Non-solid	Part-solid	Solid	Total
Non-solid	**19.22** ± **0.73**, (**18.78** ± **0.5**)	0.78 ± 0.73, (1.21 ± 0.5)	0 ± 0.0, (0.01 ± 0.1)	20
Part-solid	0.07 ± 0.3, (1.22 ± 0.5)	**17.38** ± **0.9**, (**16.38** ± **0.9**)	2.55 ± 0.8, (2.4 ± 0.8)	20
Solid	0.01 ± 0.1, (0.03 ± 0.2)	1.58 ± 1.2, (1.3 ± 1.2)	**18.41** ± **1.2**, (**18.67** ± **1.2**)	20
Total	19.30 ± 0.9, (20.03 ± 0.7)	19.74 ± 1.3, (18.89 ± 1.3)	20.96 ± 1.3, (21.08 ± 1.3)	60
**Accuracy (%)**		**Precision and Recall**		
		**Non-solid (%)**	**Part-solid (%)**	**Solid (%)**
91.7 ± 1.3		99.6 ± 0.9, 96.1 ± 1.1	88.0 ± 1.9, 86.9 ± 1.5	87.8 ± 1.8, 92.1 ± 1.1
(89.7±1.1)		(93.8 ± 0.9, 93.9 ± 1.0)	(86.7 ± 1.7, 81.9 ± 1.4	(88.6 ± 1.7, 93.4 ± 1.0)

The vertical dimension reports the radiologists’ classification, whereas the horizontal row is the classification results from the CNN model. The mean ± standard deviation statistics for the accuracy, precision and recall of the CNN model over the 10 times 10-fold cross validations are also reported below the confusion matrix.

Note: Numbers within parentheses are the results of strategy SINGLE, whereas numbers out of parentheses are the results of strategy ALL.

**Table 2:** Mean ± standard deviation summary of confusion matrixes achieved by HIST model over 10 times 10-fold cross validations on the testing nodules.

The mean confusion matrix of HIST over the cross-validation of all folds				
Rad\HIST	Non-solid	Part-solid	Solid	Total
Non-solid	**19.57** ± **0.73**, (**18.41** ± **0.7**)	0 ± 0.2, (0.06 ± 0)	0.43 ± 0.7, (1.53 ± 0.7)	20
Part-solid	3.64 ± 0.4, (2.12 ± 0.6)	**12.06** ± **1.4**, (**11.62** ± **1.0**)	4.3 ± 1.3, (6.26 ± 1.0)	20
Solid	6.23 ± 0.5, (5.39 ± 0.5)	1.46 ± 0.6, (2.91 ± 0.6)	**12.31** ± **0.8**, (**11.7** ± **0.8**)	20
Total	29.44 ± 1.8, (25.92 ± 2.4)	13.52 ± 2.6, (14.59 ± 2.8)	17.04 ± 1.6, (19.49 ± 2.3)	60
**Accuracy (%)**		**Precision and Recall**		
		**Non-solid (%)**	**Part-solid (%)**	**Solid (%)**
73.2 ± 1.8		66.5 ± 1.9, 97.9 ± 1.2	89.2 ± 1.1, 60.3 ± 1.4	72.2 ± 1.5, 61.6 ± 1.2
(69.6 ± 1.6)		(71.0 ± 2.9, 92.1 ± 1.5)	(79.6 ± 2.4, 58.1 ± 1.7)	(60.0 ± 1.9, 58.5 ± 1.8)

The vertical dimension reports the radiologists’ classification, whereas the horizontal row is the classification results from the HIST model. The mean ± standard deviation statistics for the accuracy, precision and recall of the HIST model over the 10 times 10-fold cross validations are also reported below the confusion matrix.

**Note:** Numbers within parentheses are the results of strategy SINGLE, whereas numbers out of parentheses are the results of strategy ALL.

Table 3 summarizes the Cohen kappa values comparing the CADx classifications with those made by expert radiologists. One complexity we faced in computing these comparisons, however, was the fact that the number of radiologists providing annotations differed between nodules, ranging from one to four annotations. To account for this, we divided the nodules into four groups corresponding to the number of radiologists who had annotated them. We then separately computed the kappa value for each group. As can been seen, the mean Cohen kappa values computed over all 100 folds were 0.74 (min-max range: 0.41–0.91; 95% CI: 0.62, 0.86), 0.73 (min-max range: 0.38–0.91; 95% CI: 0.60, 0.85), 0.56 (min-max range: 0.46–0.68; 95% CI: 0.50, 0.63), and 0.61 (min-max range: 0.48–0.74; 95% CI, 0.50, 0.68) for the CNN-SINGLE, CNN-ALL, HIST-SINGLE, and HIST-ALL classifications, respectively. This corresponds to substantial or near-perfect agreement^25^ with radiologists’ classification results. The inter-observer agreement between different radiologists is also reported in Table 3. As can be seen, the overall mean Cohen kappa value across all four groups was 0.74 (min-max range: 0.50–0.89; 95% CI: 0.66, 0.82), suggesting substantial agreement.

**Table 3:** Summary of agreement between radiologists and the CAD algorithms and inter radiologist annotation agreement for the classification of solid, part-solid, and non-solid over the 10 times 10-fold cross-validation.

Annotation Agreement between Radiologists and the CAD Algorithms				
	Kappa value			
	CNN SINGLE	CNN ALL	HIST SINGLE	HIST ALL
**One annotation**				
Annotation 1	0.87(0.85–0.88)	0.91(0.90–0.93)	0.68(0.67–0.68)	0.74(0.73–0.75)
**Two annotations**				
Annotation 1	0.90(0.87–0.91)	0.89(0.87–0.91)	0.61(0.59–0.63)	0.67(0.66–0.71)
Annotation 2	0.76(0.75–0.77)	0.74(0.73–0.76)	0.66(0.64–0.67)	0.71(0.70–0.74)
**Three annotations**				
Annotation 1	0.91(0.88–0.95)	0.88(0.85–0.93)	0.67(0.62–0.70)	0.71(0.68–0.75)
Annotation 2	0.66(0.63–0.70)	0.63(0.60–0.68)	0.54(0.53–0.55)	0.48(0.42–0.53)
Annotation 3	0.41(0.38–0.45)	0.38(0.35–0.43)	0.49(0.48–0.50)	0.48(0.42–0.53)
**Four annotations**				
Annotation 1	0.88(0.87–0.89)	0.88(0.87–0.89)	0.48(0.44–0.52)	0.58(0.57–0.60)
Annotation 2	0.66(0.64–0.67)	0.66(0.64–0.67)	0.46(0.42–0.49)	0.52(0.51–0.53)
Annotation 3	0.77(0.76–0.78)	0.77(0.76–0.79)	0.57(0.52–0.60)	0.64(0.62–0.66)
Annotation 4	0.55(0.53–0.56)	0.55(0.53–0.56)	0.46(0.42–0.49)	0.53(0.51–0.54)
**Inter-radiology Annotation Agreement**		**Nodule distributions over Annotation Numbers**		
**Two annotations**	Kappa value	**One annotation [33/60]**	3-class*	5-score^+^
Annotation 1 vs 2	0.71	Annotation 1	12, 12, 9	8, 4, 12, 2, 7
**Three annotations**		**Two annotations[14/60]**		
Annotation 1 vs 2	0.75	Annotation 1	7, 1, 6	7, 0, 1, 1, 5
Annotation 1 vs 3	0.50	Annotation 2	6, 3, 5	6, 0, 3, 3, 2
Annotation 2 vs 3	0.75	**Three annotations [4/60]**		
**Four annotations**		Annotation 1	0, 1, 3	0, 0, 1, 0, 3
Annotation 1 vs 2	0.78	Annotation 2	0, 0, 4	0, 0, 0, 0, 4
Annotation 1 vs 3	0.67	Annotation 3	0, 1, 3	0, 0, 1, 0, 3
Annotation 1 vs 4	0.67	**Four annotations [9/60]**		
Annotation 2 vs 3	0.89	Annotation 1	2, 3, 4	2, 0, 3, 0, 4
Annotation 2 vs 4	0.89	Annotation 2	2, 1, 6	2, 0, 1, 0, 6
Annotation 3 vs 4	0.78	Annotation 3	1, 2, 6	1, 0, 2, 0, 6
		Annotation 4	3, 0, 6	3, 0, 0, 0, 6

Note: Numbers with parentheses are the min-max ranges of kappa values.

*From left to right are the numbers of non-solid, part-solid and solid nodules, respectively.

^+^From left to right are the numbers of nodules with the score of 1, 2, 3, 4, and 5, respectively.

### Regression Comparison to Radiologists’ Ratings

Table 4 summarizes the root mean square error (RMSE) between the CADx regression scores and the expert ratings for the nodules in each of the four annotation groups. More specifically, for each annotation group, we provide the mean and median RMSE value for each combination of algorithm and slice strategy. For example, we report the median RMSE value computed using the regression scores from the CNN algorithm and the ALL slice selection strategy as “CNN-ALL-Median.” The mean RMSE values across the annotation groups of CNN-ALL-Mean, CNN-ALL-Median, HIST-ALL-Mean and HIST-ALL-Median were 0.92 (95% CI: 0.90, 0.93), 0.89 (95% CI: 0.87, 0.91), 1.01 (95% CI: 0.99, 1.02) and 1.01 (95% CI: 0.99, 1.02), respectively. The mean RMSE between the radiologists’ ratings was 0.99 (95% CI: 0.98, 1.00).

**Table 4:** Root Mean Squared Error statistics comparing the CAD algorithms and annotated scores in the four nodule groups.

The Root Mean Squared Error between the CADs' and Radiologists' Scores				
	Group 1	Group 2	Group 3	Group 4
CNN-ALL-Mean	0.92 ± 0.16	0.91 ± 0.12	0.92 ± 0.23	0.92 ± 0.14
HIST-ALL-Mean	1.01 ± 0.13	1.01 ± 0.14	1.01 ± 0.21	1.01 ± 0.12
CNN-ALL-Median	0.91 ± 0.14	0.89 ± 0.13	0.89 ± 0.21	0.89 ± 0.15
HIST-ALL-Median	1.00 ± 0.15	1.01 ± 014	1.01 ± 0.18	1.01 ± 0.13
CNN-SINGLE	0.93 ± 0.14	0.95 ± 0.15	0.94 ± 0.20	0.93 ± 0.13
HIST-SINGLE	1.00 ± 0.15	1.00 ± 0.14	0.99±017	1.01 ± 0.15
Between-Grps		1.07 ± 0.00	1.05 ± 0.3	0.9 ± 0.20

The nodules Group 1, 2, 3 and 4 have 1, 2, 3 and 4 annotations, respectively. The last row “Between-Grps” reports the statistics of inter-radiologists’ rating scores over all 4 groups. The pairs tagged “ALL-Mean” are the results with the ALL strategy using the mean of the regressed scores over all member slices of a nodule. The pairs tagged “ALL-Median” are the results with the ALL strategy using the median regressed scores over all member slice of a nodule. The pairs tagged “SINGLE” are the results with the SINGLE strategy.

**Figure 2:**
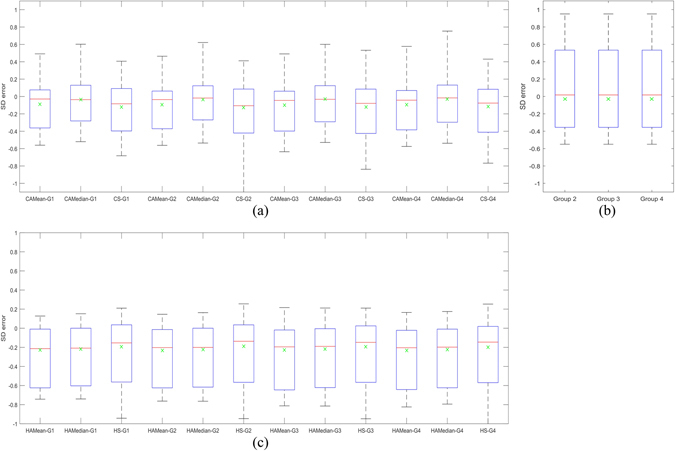
(**a**) Box-plots of signed differences (SDs) between the CNN results of the four annotation groups with respect to the three strategies of ALL-mean, ALL-median and SINGLE. ALL and SINGLE are the slice selection strategies, whereas the mean and median suggests the method of integration the prediction scores with the ALL strategy. (**b**) Box-plots of SDs for the annotation groups 2-4, each group corresponding to the number of radiologists who provided annotations. (**c**) Box-plots of SDs between HIST results of the four groups with respect to the ALL-mean, ALL-median and SINGLE strategies. In all box-plots, the mean value of each performance distribution is also illustrated with green-cross makers. The tag “CAMean” stands for CNN-ALL with the mean score over all member slices of a nodule, the tag “CAMedian” stand for CNN-ALL with the median score of all member slices of a nodule, “CS” suggests the CNN method with SINGLE strategy, while “G1” stands for the 1^st^ group, “G2” stands for the 2^nd^ group, and so on. For HIST, only the first capital letter is replaced with ‘H’, while the other letters remain hold the same meaning.

Figure 2 demonstrates the box-plots of the signed difference (SD) distributions between the CADx methods (including ALL and SINGLE strategies) and the annotation ratings in the four groups, where the red lines delimit the median of SD measures in each comparing pair, and the green crosses indicate the SD mean values. Referring to Fig. 2(b), the first and third quarters of inter-observer SD values were around −0.39 and 0.45, with median SD nearly close to zero. For all CNN regression results, the averaged first and third quarters of SD values were around −0.35 and 0.15, with the median SD around −0.1. For all HIST regression results, the first and third quarters of SD values were around −0.6 and 0, with the median SD around −0.2. It can be observed from Fig. 2 that the HIST regression scores were much less than the radiologists’ ratings. We also perform two-sample *t*-tests to evaluate the significance of differences between the regression scores from either CNN-ALL or HIST-ALL to the radiologists’ overall ratings. The *p*-values were found to be 0.4967 and 0.4191 respectively, suggesting that the CNN scores were not significantly different from the radiologists’ ratings. Referring to Table 4 and Fig. 2, it can be found that slicing strategy ALL performed a little better than strategy SINGLE for regression, whereas the performances of two ALL strategies (CNN-ALL-Mean and CNN-ALL-Median) were similar.

## Discussion and Conclusion Remarks

The radiological identification of non- and part-solid pulmonary nodules is essential for the diagnosis and subsequent management of pulmonary adenocarcinoma^1, 3, 11, 15, 16, 26^. Despite its importance, however, the radiological task of distinguishing between non-, part-, and solid nodules has proved difficult, with intra- and inter-observer variation in nodule classification ranging from moderate to significant^18–20^. Quantitative methods in CADx systems promise to improve the consistency and reliability of pulmonary nodules classification. In this paper, we have described an accurate new CADx algorithm built upon a convolutional neural network.

The model presented in this paper demonstrated both internal consistency as well as concordance with expert opinions. Across all test nodules, we report a mean Cohen kappa value of 0.74, suggesting substantial agreement at a level consistent with the state of the art^20^. Further, our CNN achieved a similar level of agreement with radiologist annotations in both classification and score regression tasks. Of particular interest, for the regression comparison, the CNN scores computed by our model demonstrated in some cases lower variance than the inter-observer ratings amongst the practicing radiologists themselves. This is likely because our CNN was trained using labels provided from more than 10 radiologists^27^ with various background and experience, effectively enabling the model to fuse the annotation knowledge across various radiologists to attain less scoring variation.

Our implementation of the established HIST method also achieved satisfactory classification performance, though not matching the performance of the CNN. In addition, the HIST method was greatly outperformed by CNN on the regression tasks. The particularly poorer relative performance of the HIST method versus the CNN method on regression tasks despite reasonable classification performance may suggest that our CNN method has a greater general capacity to learn finer grained visual features of the pulmonary nodules. If so, this finer capacity may ultimately prove important for the ultimate task of executing differential diagnosis of adenocarcinomas^14^. Finally, as described above, the HIST method requires image segmentation, introducing a potential source of uncertainty.

In both classification and regression tasks, our models achieved the greatest performance with the ALL slice selection strategy rather than the SINGLE slice selection strategy. We expect that this is due to geometric eccentricity of the solid portions of the nodules: since the SINGLE strategy only utilizes the median slice from each orthogonal view of the nodule, this approach will be unable to characterize any solid portions of the nodule that extend haphazardly in one or more narrow directions. In such cases, a single slice in each direction will not be representative of the nodule as a whole, and the SINGLE strategy will be insufficient. For this reason, we suggest that including additional CT slices of the nodule can be helpful attenuation characterization tasks.

In summary, we have here reported the development of a CNN-based CADx for three-class and five-score categorization of pulmonary nodules. We have also conducted a comprehensive performance comparison of this CNN-based method to an established histogram-based approach. In each case, our CNN model outperformed the HIST method without the need of image segmentation^28^, achieving substantial agreement with the scores and classifications provided by expert radiologists. This suggests that convolutional neural networks may improve the performance of CADx systems. When successfully implemented, this may be useful at a range of scales, from serving as a second opinion for practicing radiologists considering biopsies of individual nodules, or to facilitate the acceleration or even automation of nodule classification over large data sets^20^.

## Materials and Methods

### Dataset and Nodule Annotation

All methods were carried out in accordance with approved guidelines, with informed consents obtained from all subjects. We utilized data from the Lung Image Database Consortium (LIDC)^27^ to train and test our classification and regression schemes. The dataset includes thoracic CT scans of 1,010 patients from seven academic medical centers in the United States. Data was collected under appropriate local IRB approvals at each of the seven academic institutions (Weill Cornell Medical College, University of California, Los Angeles, University of Chicago, University of Iowa, University of Michigan, MD Anderson Cancer Center and Memorial Sloan-Kettering Cancer Center), see details in ref. ^27^. The slice thickness of the CT scans in the LIDC dataset ranges from 1.25mm to 3mm, and the slice spacing varies between 0.625mm to 3.0mm. Both standard-dose CT scans and low-dose CT scans are included, a subset of which are contrast-enhanced while the others are non-contrast. 12 radiologists from 5 medical centers were involved in the annotation process. Each thoracic CT scan was annotated by four of the twelve radiologists under a rigorous two-phases image reading process (blinded and unblinded). For nodules with diameters larger than 3 mm, each radiologist was asked to define the nodule boundaries and give the rating scores with respect the characteristics of “Subtlety”, “Calcification”, “Sphericity”, “Margin”, “Spiculation”, “Texture”, and “Malignancy”. Of these, the “Texture” characteristics reflects the internal density, with the annotation corresponding to a pulmonary nodule attenuation rating score ranging from 1 to 5. Nodules with a “Texture” score of 1 were identified as non-solid/ground glass opacity, nodules scored as 3 were taken as partial/mixed solid nodules, and nodules rated with score 5 were considered completely solid. Figure 1 (b) depicts several nodules that were rated as the scores of 1, 3 and 5, respectively. The left two nodules are classified as non-solid with annotated score of 1, the middle two nodules are classified as part-solid scored as 3, and the right two nodules are classified as solid scored as 5.

Richly annotated and publically available datasets of pulmonary nodule CT scans such as LIDC provide a quantitative reference for clinical image reading, a resource for residents and medical students learning to diagnose images, and a gold standard dataset for computational researchers seeking to train models such as ours. Nevertheless, the dataset is not without its challenges and limitations. First, as depicted in Fig. 1 (a), discernible classification disagreements are apparent in some cases among the radiologist ratings. In addition, high variation of shape and appearance of the nodules in each category impose additional difficulty for the development of a computerized scheme.

### Computerized Categorization of Solid, Part-Solid and Non-Solid Nodules and Machine Learning Model

To tackle the issue of high variation in the shape and appearance of nodules, we exploited the deep learning model of convolutional neural networks^29, 30^ to automatically discover useful computational features. Unlike the conventional CAD scheme that requires explicit feature engineering to characterize nodule morphology and texture, the CNN employs a hierarchical architecture of connected layers of neurons to automatically extract the nodule features that are relevant to the machine learning task. This structure mimics the architecture of the visual perceptual system of animals, and could be extended beyond our current task detect and rate previously unseen nodules in CT scans.

**Figure 3:**
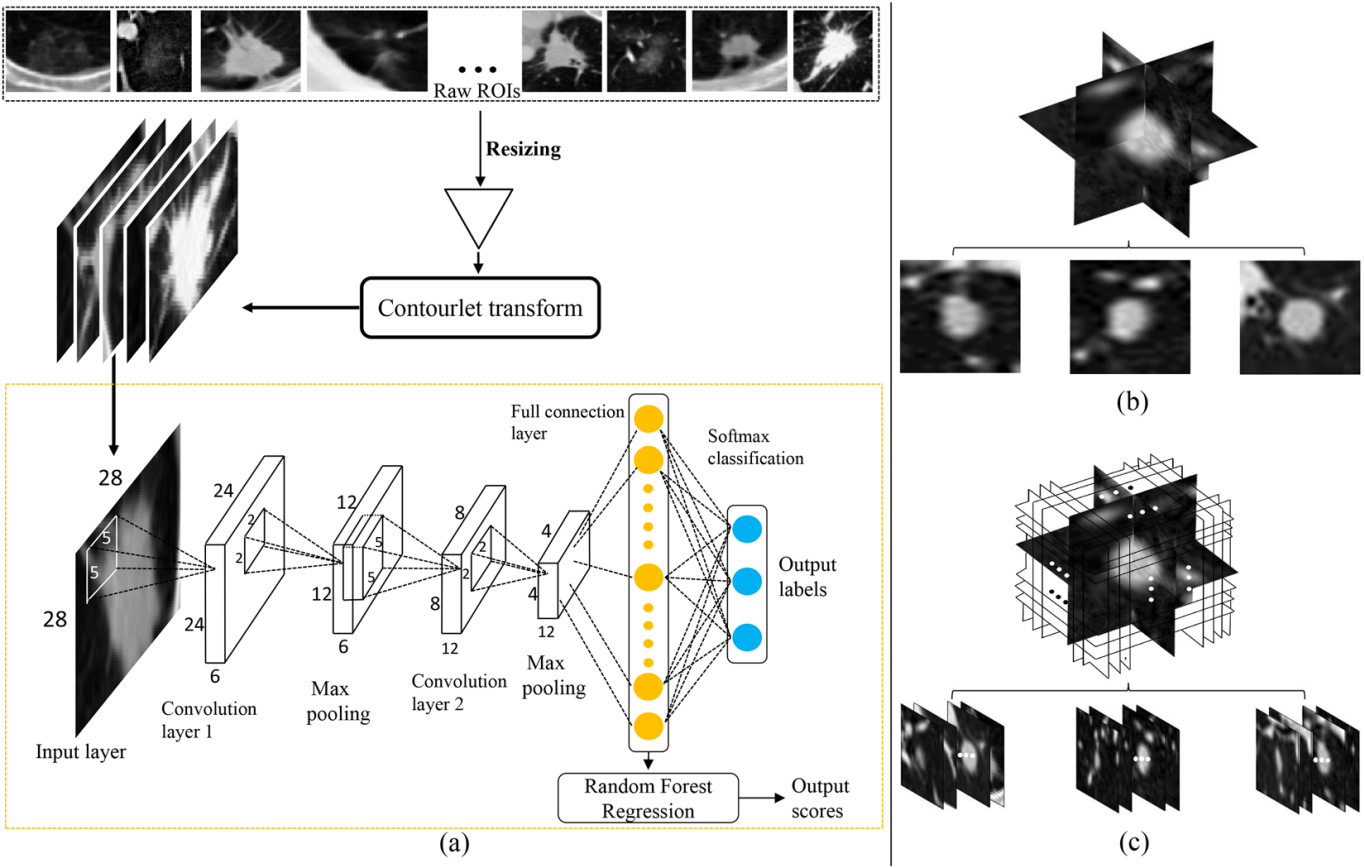
(**a**) Flow-chart of our deep-learning-based CADx training framework. The pixels in nodule ROIs are treated as input neurons for the CNN network for feature learning. (**b**) The SINGLE strategy using multi-view sampling. (**c**) The ALL strategy using multi-view sampling.

In this study, we exploited two computerized texture analysis schemes, classification and regression, to automatically evaluate nodule attenuation in CT scans using the high hierarchical features learned from a CNN model. For the classification task, we categorized the pulmonary nodules into the non-solid, part-solid and solid classes. Specifically, nodules scored as 1 and 2 were labeled as non-solid, nodules with the score of 3 were labeled as part-solid, and the remaining nodules of scores 4 and 5 were identified as solid. If a nodule was rated by multiple radiologists, we simply averaged the radiologists’ rating scores for the training and testing, as was done in previous studies^31^. We randomly selected 190 nodules for each of the three classes (totally 570 nodules) for the training and testing of CNN model. Along with the original CT image contents, we also incorporated the contourlet transform^32^ to decompose the CT images into several components to provide more diverse image cues for the CNN model as data augmentation. For the regression task, we used the features computed at the final fully-connected layer of the trained CNN as input into a random forest regression scheme to automatically rate the nodules with the semantic scoring range from 1 to 5^33^. The network architecture can be found in the Fig. 3(a), and was implemented using the Caffe^34^ toolbox. Specifically, our CNN was constructed with two convolutional layers (5 × 5 kernel size with stride 2), each followed by a max-pooling layer (2 × 2 kernel size with stride 2). The number of feature maps in the first and the second convolutional layer were 6 and 16, respectively. A fully-connected layer was then connected to the pooling-2 layer, and further followed by a Softmax activation layer for the prediction of class label. The base learning rate was set as 0.05 and fixed throughout the training process.

### Training and Testing of CNN model

**Figure 4:**
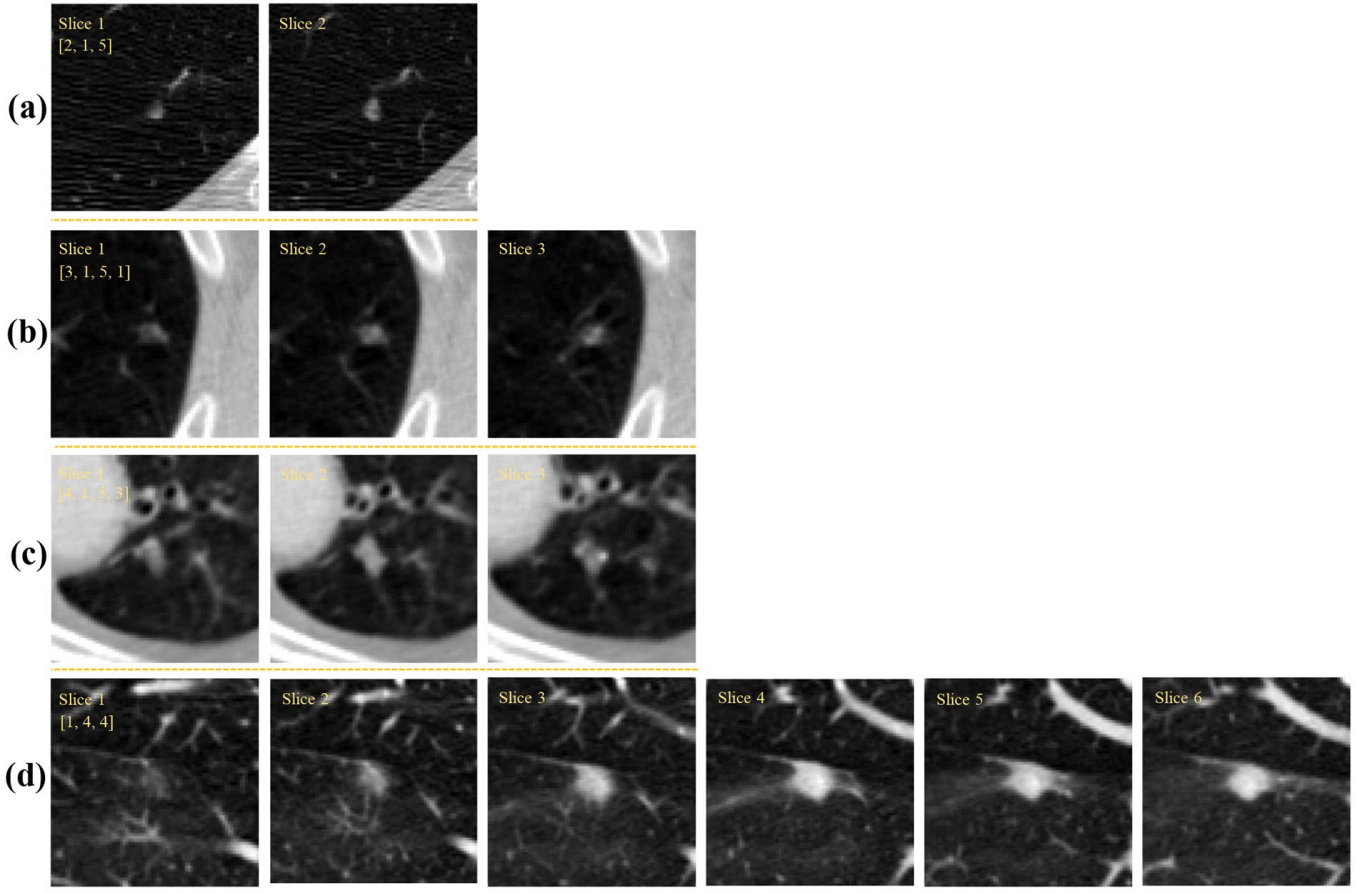
Difficult nodules contained in more than one slice. The scores of each nodule rated by radiologists are shown in the upper portion of the first slice ROI of each nodule with yellow numbers. The slice numbers of nodule cases (**a**,**b**,**c** and **d**) are 2, 3, 3 and 6, respectively.

Due to the high variation of slice thickness (1.25–3mm) in the LIDC CT data, the training and testing of the CNN model was based on 2D ROIs to avoid the direct computation of 3D features. The 2D ROIs for each nodule were based on the nodule contours drawn by radiologists, with the expanding offsets of 10 pixels added to the ROI to include more anatomic context for CNN. Since a pulmonary nodule with diameter larger than 3 mm can be depicted with more than 1 slice of the CT scan, as shown in Fig. 4, it is generally unknown how many member slices should optimally be included to best represent a nodule in the training of machine learning models. In this study, we implemented two slice selection strategies: SINGLE and ALL^7^, with samples obtained in each case from transversal, coronal and sagittal views^35^. For the SINGLE strategy, only the median slices of the three views were selected as the representative cues for training and testing, see Fig. 3(b). For the ALL strategy, all slices from the three orthogonal views were selected as representative samples for training and testing, see Fig. 3(c). In training the CNN model, we randomly shuffled the 2D ROIs irrespective of the concept of nodules.

For testing, the score or class was determined by combining the results from all the ROIs selected for a given nodule via the slicing strategy. In the case of the SINGLE slice selection strategy, we simply computed the average of the scores or classes from the three median slices the strategy selected for each ROI. For the ALL strategy classification task, we likewise computed consensus classifications by averaging the results from all the ROI slices. In the case of ALL strategy regression, we implemented two schemes to compute final scores: First, we computed what we termed the ALL-Mean consensus score, which was, as above, the average over all slices irrespective of orientation. Second, we computed the ALL-Median consensus score, which we computed for each nodule by first computing the median score from the slices in each of the three orientations (coronal, sagittal, axial) and then averaging the three orientation-specific results.

### Performance Evaluation and Statistical Analysis

To avoid data dependence, we utilized 170*3 nodules for training and validation, and present all results based on independent set of 20 nodules per class for testing that were not involved in the training or validation steps. The experiment was performed using 10 rounds of 10-fold cross validation, where each round involved an independent random selection set of 170*3 nodules from the 190*3 nodules. For each fold of one 10-fold cross validation, 153 nodules were used for training and validation, whereas 17 nodules were used for testing.

To assess our model in context of the previous state of the art methods, we also implemented a histogram-based analysis as described by Jacobs *et al*.^20^, denoted hereafter as HIST. In this approach, nodules are segmented and binned into subregions, for which various summary statistics are computed and used to train a classifier. We recapitulated this analysis including the computation of bin statistics for each nodule including entropy, standard deviation, mean height, the height of the bin with most voxels, and the 5%, 25%, 75% quantiles of voxels. We then applied a *k*-nearest neighbor (KNN) classifier with a neighbor parameter *k* of 12. To ensure a fair comparison of HIST with our own method, we ensured that training and the testing was computed using the same data partition with respect to each fold when running the 10 times 10-fold cross-validation. Another important consideration when evaluating the HIST method was the choice of method used to process the nodules prior to the statistical analysis. In previous works, nodule regions were defined using a segmentation algorithm, which was originally designed for solid nodule segmentation^36^. However, segmentation algorithms have the potential to introduce error, especially when blindly applied to new datasets. Although we considered using a segmentation approach based on non-solid tissue Hounsfield Unit (HU) range^20^, the available HU range had been defined by a previous phantom study^37^, which may not generalized to real cases. Fortunately, the LIDC dataset contains manual nodule delineations performed by expert radiologists, which enabled us to directly utilize these gold standard segmentations without concern for algorithm-induced uncertainty.

We computed confusion matrices to illustrate the classification performance of our algorithm and the HIST method. In each case, “true” labels (denoted as “Rad” in the confusion matrices) were defined based on the nodule’s first listed radiologist annotation in the dataset. However, we noted that in the LIDC dataset, a pulmonary nodules contain ratings from more than 10 independent radiologists from different institutions in the United States, with any given nodule being annotated by either one, two, three or four distinct radiologists. Therefore, to avoid systematic bias, we divided the nodules into four groups corresponding to the number of annotations each nodule received. We then reported the performance of our algorithms both overall as well as on a stratified basis in which we only reported accuracy on groups of nodules where each nodule had been annotated by the same number of radiologists (though not necessarily the same radiologists).

Finally, we computed statistical metrics to compare the level of agreement between our classification and regression performance and the assessments of the radiologists. To this end, for each of the four annotation groups, we used the Cohen kappa statistic^25, 38^ to quantify the classification agreement between the computer methods and the radiologists, as well as among the radiologists themselves. Similarly, to compare the regression scores computed by our algorithms with the evaluation of the radiologists, we computed the Root Mean Squared Error (RMSE) metrics and signed difference (SD) statistics.

### Data availability statement

The data sources are public and openly available to everyone with the URL: https://wiki.cancerimagingarchive.net/display/Public/LIDC-IDRI.
